# A Grand Challenge of Factors Influencing Lung Health

**DOI:** 10.3389/fmed.2014.00011

**Published:** 2014-05-01

**Authors:** Laurent P. Nicod

**Affiliations:** ^1^Service of Pneumology, Department of Medicine, Centre Hospitalier Universitaire Vaudois, Lausanne, Switzerland

**Keywords:** lung health, lung diseases, lifestyle factors, environmental factors, biomarkers

Respiratory diseases whether communicable or non-communicable, acute or chronic impact on health of millions of people.

Several research areas, strongly linked to the pathogenesis of respiratory diseases have been identified as priorities overarching different specialties (1, 2). Some are related to early origin of lung disease. Lifestyle choices have obvious consequences as well as environmental factors related to inhaled particles or pathogens impacting the integrity of the airways, or of our microbiote with dysbiose. Lung defenses are important to preserve our lungs from aggression. Their changes in life are important allowing more vulnerability early in childhood as well as late in life. Lung diseases are more frequent with aging and this represents several challenges.

The early origin of lung diseases should be better analyzed to understand lung development and prevent diseases impacting adulthood (3). Prenatal factors are known to influence lung health later in life with both nutritional deficiencies and maternal smoking already recognized as having epigenetic and trans-generational effects (4). Life expectancy of several congenital defects has improved as infants with cystic fibrosis or neuromuscular disease benefit from early interventions with new targeted biological approaches (5). Effective prevention of several early infections is important, in order to reduce incidence of premature death or bronchiolitis with long term sequelae. The role of microbiote for the education of the immune system appear an important challenge, to prevent allergies to common allergens in the form of rhinitis or asthma of varying severity, often persisting into adulthood (6, 7).

Lifestyle can impact lung health markedly. It is established that nutritional deficiencies impair lung growth, favor infections, and can decrease the ability to control inflammatory processes due, for instance, to lack of antioxidative factors (8, 9). A decrease of physical activity, often combined with obesity, impacts on disorders such as asthma. Obesity also leads to obstructive sleep apnea syndrome (OSAS) (10). The overall cardiovascular and metabolic consequences of OSAS are now recognized to be significant and add to the overall burden of diseases. The physiologic process involved need to be better understood. Inhaled particles, volatile organic compounds, active and passive smoking are major factors in the etiology of chronic obstructive lung diseases (COPD) (11). Asthma and COPD are the most prevalent respiratory diseases placing a major burden on health care systems. The local and systemic effects of these diseases still deserve intensive research for prevention and new targeted therapies.

Among the environmental factors, carcinogens found in tobacco smoke are known to be abundant and contribute to one of the two most frequent cancers, which is lung cancer, the first in men and the second in women. Other carcinogens include the radioactive element radon, which may be released naturally from the granite below houses (12), or asbestos extracted from mines and used for building insulation. All such compounds need to be identified and environmental exposure to them minimized.

Lung defenses and infections are at the crossroads of several specialties. The integration of our knowledge is important, as acute lower respiratory infections (such as bacterial and viral pneumonia, influenza, and respiratory syncytial virus infections) are the third most frequent cause of death worldwide, accounting for 4.25 million deaths each year. Underlying chronic lung diseases often alter lung defenses and influence the type infection found (13, 14). Several chronic lung diseases like interstitial lung disease, cystic fibrosis, or asthma among other chronic obstructive disease can be markedly exacerbated by the occurrence of infections (15). Preventions of infections and control of inflammation induced in such circumstances are challenges for the future. New vaccines and antiviral agents are also needed.

Aging causes a reduction in the gas-exchange surface of the lungs, which may lead to a reduced capacity to oxygenate blood. The airways become more collapsible, worsening any underlying obstructive disease (16). Lung defenses are altered and infections increased in frequency (17). Prevention of undue inflammation related to environmental factors might decrease the effects of this natural decline in airway function. Lung injury related to inhaled particles or to infections can produce fibrotic processes related to defects in natural repair, to alterations in lung matrix with consequences in diseases like lung fibrosis and/or to a higher incidence of autoimmune diseases among older people (18). The immune process lead not only to scaring of distal airways, but also in some patients to vascular narrowing and to a higher incidence of pulmonary arterial hypertension with age (19). With aging, dysregulation of lung tissue regeneration by progenitor cells can occur, leading to various thoracic malignancies. Malignancy is also related to carcinogens or to genetic factors and identification of these may result in more individualized diagnostic screening and more personalized treatment.

New technologies are becoming available for improved imaging, with more specific biomarkers and for more precise targeting of metabolic pathways. These should allow earlier and more specific diagnosis, as well as better targeted and personalized treatments. One can hope that these new developments will decrease side effects of current therapies, improving disease outcomes and promote more healthy aging. Frontiers in pneumology should reflect all these new developments.

## Conflict of Interest Statement

The author declares that the research was conducted in the absence of any commercial or financial relationships that could be construed as a potential conflict of interest.
